# Recent Advances in β-Ga_2_O_3_–Metal Contacts

**DOI:** 10.1186/s11671-018-2667-2

**Published:** 2018-08-22

**Authors:** Ya-Wei Huan, Shun-Ming Sun, Chen-Jie Gu, Wen-Jun Liu, Shi-Jin Ding, Hong-Yu Yu, Chang-Tai Xia, David Wei Zhang

**Affiliations:** 1 State Key Laboratory of ASIC and System, School of Microelectronics, Fudan University, Shanghai, 200433 China; 2 Division of Microelectronics, School of Science, Ningbo University, Ningbo, 315211 China; 3 Department of Electrical and Electronic Engineering, Southern University of Science and Technology, Shenzhen, 518055 China; 4 Key Laboratory of Materials for High Power Laser, Shanghai Institute of Optics and Fine Mechanics, Chinese Academy of Science, Shanghai, 201800 China

**Keywords:** β-Ga_2_O_3_, Contacts, Metal stacks, Intermediate semiconductor layer

## Abstract

Ultra-wide bandgap beta-gallium oxide (β-Ga_2_O_3_) has been attracting considerable attention as a promising semiconductor material for next-generation power electronics. It possesses excellent material properties such as a wide bandgap of 4.6–4.9 eV, a high breakdown electric field of 8 MV/cm, and exceptional Baliga’s figure of merit (BFOM), along with superior chemical and thermal stability. These features suggest its great potential for future applications in power and optoelectronic devices. However, the critical issue of contacts between metal and Ga_2_O_3_ limits the performance of β-Ga_2_O_3_ devices. In this work, we have reviewed the advances on contacts of β-Ga_2_O_3_ MOSFETs. For improving contact properties, four main approaches are summarized and analyzed in details, including pre-treatment, post-treatment, multilayer metal electrode, and introducing an interlayer. By comparison, the latter two methods are being studied intensively and more favorable than the pre-treatment which would inevitably generate uncontrollable damages. Finally, conclusions and future perspectives for improving Ohmic contacts further are presented.

## Introduction

Recently, gallium oxide (Ga_2_O_3_) has been considered as a promising candidate for preparing high-power and high-efficiency devices by virtue of its excellent material properties [1–3]. Ga_2_O_3_ has five different polymorphs, designated as α-Ga_2_O_3_, β-Ga_2_O_3_, γ-Ga_2_O_3_, δ-Ga_2_O_3_, and ε-Ga_2_O_3_, among which β-Ga_2_O_3_ is the most thermodynamically stable and has been extensively studied [4]. With ultra-wide bandgap of 4.6–4.9 eV [5–7], the theoretical breakdown electric field (*E*_br_) of 8 MV/cm for β-Ga_2_O_3_ is about three times larger than that of SiC or GaN [8–10], which enables β-Ga_2_O_3_-based devices to handle gigantic switching voltages. The suitability of semiconductors for power device applications is usually evaluated by Baliga’s figure of merit (BFOM) [11]. The BFOM of β-Ga_2_O_3_ is almost triple that of SiC and GaN, reducing the conduction loss significantly [3, 12–14]. Moreover, the saturation electron velocity is theoretically estimated to be around 2 × 10^7^ cm/s, making it alluring for high-frequency operations [15–20]. Another distinctive interest of β-Ga_2_O_3_ among wide-bandgap semiconductors is that high-quality single crystals can be synthesized cost-effectively by using melt growth techniques [21–24]. In addition, high-quality n-type β-Ga_2_O_3_ epitaxial films can be realized by precisely doping with Sn, Si, Ge, and Mg, and the obtained electron density ranges from 10^16^ to 10^19^ cm^−3^ [25–28]. Because of the abovementioned advantages over other wide-bandgap semiconductors, β-Ga_2_O_3_ shows its capabilities to be a promising material for power electronics as well as extreme environment (high temperature, high voltage, and high radiation) [29–31] electronics.

Many promising β-Ga_2_O_3_ devices have been reported, including Schottky barrier diodes [32], MOSFETs [1–3], and various types of solar-blind photodetectors [33, 34]. Among these devices, MOSFETs are the most prevailing configuration for radio frequency and high-power operation [35], giving full play to its high *E*_br_ and BFOM. However, one of the challenges for β-Ga_2_O_3_ application in MOSFET devices is the difficulty in forming Ohmic contacts compared with narrow-bandgap semiconductors [36]. Usually, an excellent Ohmic contact between the semiconductor and the metal electrode is essential for high-performance semiconductor devices [37, 38]. Low-resistance contacts could reduce the voltage drop on the contact and consequently increase the voltage across the channel, securing the designed current density and high switching speeds. Furthermore, low-resistance contacts contribute to reducing heat generation which could aggravate the self-heating effect.

In consequence, the fabrication of high-quality Ohmic contacts is a prerequisite for achieving high-performance devices. In this review, we start with fundamental concepts of metal/semiconductor contacts. In the “Approaches to Ohmic Contacts” section, a summary of recent significant advances on Ohmic contacts to β-Ga_2_O_3_ is presented, and approaches to Ohmic contacts are discussed and analyzed. Finally, some perspectives are provided for improving Ohmic contacts to β-Ga_2_O_3_ in the future.

## Basic Physics of Ohmic Contacts

**Fig. 1:**
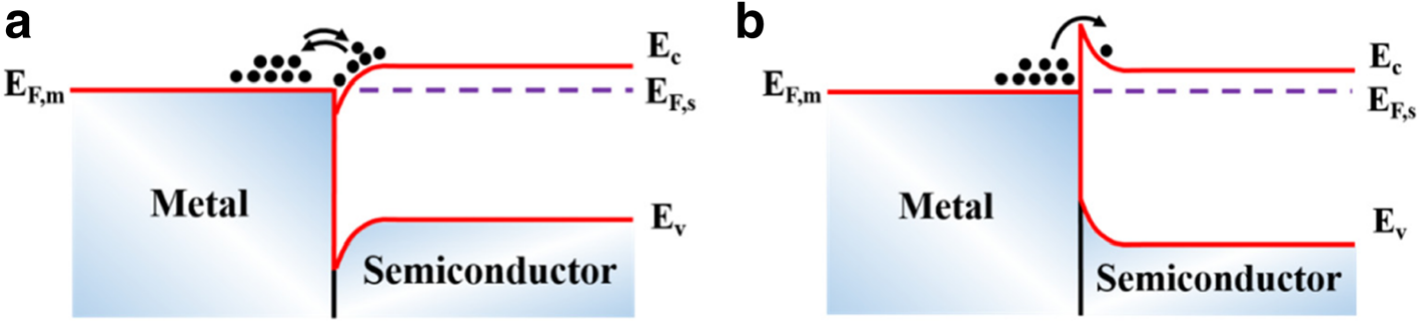
Schematic illustrations of **a** Ohmic contacts and **b** Schottky contacts. *E*_*C*_, *E*_*V*_, *E*_*F*, *m*_, and *E*_*F*, *s*_ are the energy levels of the conduction band edge, valence band edge, Fermi energy of metal and semiconductor, respectively

An Ohmic contact is a metal/semiconductor junction in which there is no barrier at the interface impeding the transport of carriers, as illustrated in Fig. 1a. On the contrary, an energy barrier existing at the interface will inhibit the carrier transport between the metal and semiconductor, as is evident from Fig. 1b. Notably, the contacts formed between wide-bandgap semiconductors and metals are always Schottky. Thus, the contact resistance normally depends on the metal/semiconductor Schottky barrier height (SBH) Φ_*B*_. For an n-type semiconductor, it obeys the equation:

1 $$ {q\Phi}_B={q\Phi}_m-{\chi}_s $$

where Φ_*m*_ is the work function of the metal and *χ*_*s*_ is the electron affinity of the semiconductor.

**Fig. 2:**
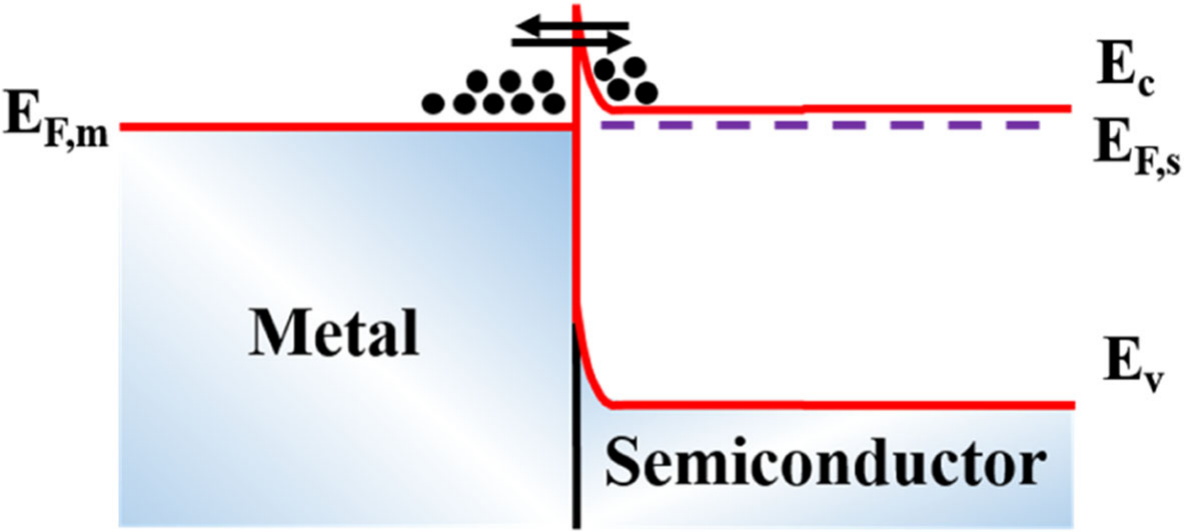
The energy band diagram at the metal/semiconductor interface with highly doped semiconductors

As depicted in Eq. (1), it is important to reduce the SBH to form the Ohmic contact. In addition, high doping in semiconductors could facilitate the formation of Ohmic contacts, e.g., for heavily doped semiconductors (N_D_ > ~ 10^18^ cm^−3^), the barrier will become narrow enough and allow the electrons directly to tunnel through the interface due to significant band bending of the conduction band [39], as shown in Fig. 2. Nevertheless, the doping levels that can be achieved in β-Ga_2_O_3_ are usually below of what can be obtained in Si, as is the case with other wide semiconductors. Other than that, the surface states also play an important role in the formation of Ohmic contacts which are frequently defined as regions of high-rate recombination. Those middle bandgap defect levels induced by the surface states are able to help the carriers transport. This implies that a good Ohmic contact can be formed by introducing proper surface states into semiconductors [40–43].

An electrical quantification of the contact characteristics is necessary to evaluate the quality of contacts. Currently, the specific contact resistivity *ρ*_*C*_ is one of the commonly used parameters to access the performance of Ohmic contacts, typically expressed in Ω ∙ cm^2^ [44]. The specific contact resistivity is a very useful quantity which is independent of the contact geometry and refers to the metal/semiconductor interface only. So far, the lowest *ρ*_*C*_ of 4.6 × 10^−6^ Ω ∙ cm^2^ was reported for Ti/Au contacts to β-Ga_2_O_3_ [45]. Wong et al. also obtained a low *ρ*_*C*_ of 7.5 × 10^−6^ Ω ∙ cm^2^ with Ti/Au contacts [46]. Up to now, many efforts have been devoted to obtain the contacting with low *ρ*_*C*_, and the typical values for specific contact resistivities spread over a range of 10^−5^–10^−6^ Ω ∙ cm^2^ for good Ohmic contacts [36].

## Approaches to Ohmic Contacts

To date, investigations on the intrinsic properties of β-Ga_2_O_3_ mostly have been carried out on its MOSFET structure, in which two kinds of the channel synthesis method are usually adopted. One is the micromechanically exfoliated flake (nanomembrane); the other is the epitaxial β-Ga_2_O_3_ film on its native substrate, as summarized in Table 1.

**Table 1:** The comparison of reported work about β-Ga_2_O_3_ MOSFETs

Channel	Carrier density/cm^−3^	Device structure	Dielectric (method)	Process	S/D metal electrode
Flake [19]	5.5 × 10^17^	BG (D-M)	285-nm SiO_2_	Tube annealing	Ti/Au
Flake [88]	3 × 10^17^	BG (D-M)	300-nm SiO_2_	–	Ti/Au
Flake [89]	3 × 10^17^	TG (E-M)	42-nm HfO_2_(ALD)	–	Ti/Au
Flake [55]	3.7 × 10^17^	BG (D-M)	300-nm SiO_2_	RTP	Ti/Au
Flake [52]	2.7 × 10^18^	BG (D/E-M)	300-nm SiO_2_	Ar plasma bombardment	Ti/Al/Au
Flake [50]	8 × 10^18^	BG (D/E-M)	300-nm SiO_2_	Ar plasma bombardment	Ti/Al/Au
Sn-doped epilayer [12]	3 × 10^17^	TG (D-M)	20-nm Al_2_O_3_(ALD)	Si^+^ implantation(S/D) + RIE + RTP	Ti/Au
UID epilayer [57]	5 × 10^19^	FP (D-M)	20-nm Al_2_O_3_(ALD)	Si^+^ implantation (channel + S/D) + RIE + RTP	Ti/Au
Sn-doped epilayer [90]	6.34 × 10^15^	TG (D/E-M)	20-nm SiO_2_(ALD)	RIE + RTP	Ti/Au
Sn-doped epilayer [13]	4.8 × 10^17^	TG (E-M)	20-nm Al_2_O_3_(ALD)	RTP	Ti/Al/Ni/Au
Sn-doped epilayer [63]	2.3 × 10^17^	WG (E-M)	20-nm Al_2_O_3_(ALD)	RTP	Ti/Al/Ni/Au
UID epilayer [46]	< 4 × 10^14^	TG (E-M)	50-nm Al_2_O_3_(ALD)	Si^+^ implantation(S/D) + RIE + RTP	Ti/Au
Sn-doped epilayer [53]	2 × 10^17^	TG (D-M)	20-nm SiO_2_ (PEALD)	Spin-on-glass doping + RTP	Ti/Au
Ge-doped epilayer [21]	4 × 10^17^	TG (D-M)	20-nm Al_2_O_3_(ALD)	RTP	Ti/Al/Ni/Au
UID epilayer [64]	–	GR (E-M)	20-nm SiO_2_(ALD)	Highly doped epitaxial cap layer on S/D + RIE + RTP	Ti/Al/Ni/Au

*BG* bottom gate, *TG* top gate, *DG* double gate, *FP* field plated, *WG* wrap gate, *D-M* depletion mode, *E-M* enhancement mode

Normally, exfoliated β-Ga_2_O_3_ flakes could be transferred to any substrates conveniently and cost-effectively. It is found that the material properties of β-Ga_2_O_3_ flakes would not degenerate during the exfoliation as evidenced by Raman spectroscopy and atomic force microscopy [19], meaning that the performance of MOSFETs based on the exfoliated flakes is comparable to that based on epitaxial layers. Due to these advantages, this method is recommended to study the electrical characteristics consisting of the density of interfacial defects, breakdown voltage, surface optical phonon scattering [47–49], and thermal property, i.e., self-heating effect [50, 51].

As summarized in Table 1, methods employed to improve Ohmic contacts could be generally categorized into three types: (1) pre-treatment, (2) post-treatment, and (3) multilayer metal electrode. Besides, introducing an interlayer can also obtain superior Ohmic contacts which is not shown in Table 1.

### Pre-treatment

**Fig. 3:**
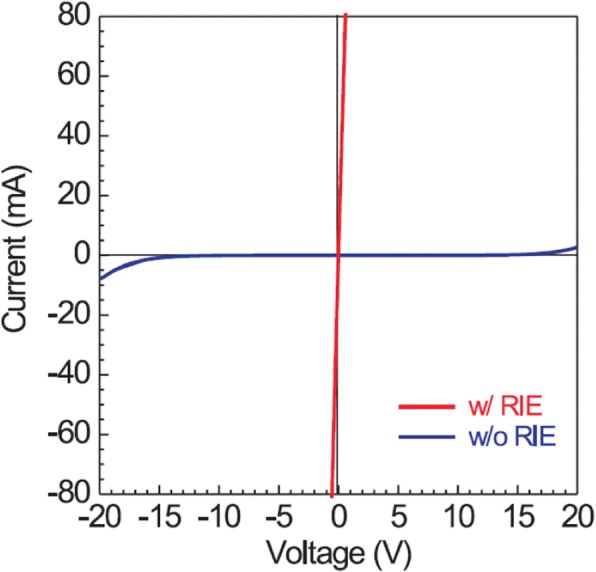
(Color online) I–V curves measured between two contacts (as-deposited Ti/Au) fabricated with and without RIE treatment on n-Ga_2_O_3_ substrates. Reproduced from Ref. [1]

**Fig. 4:**
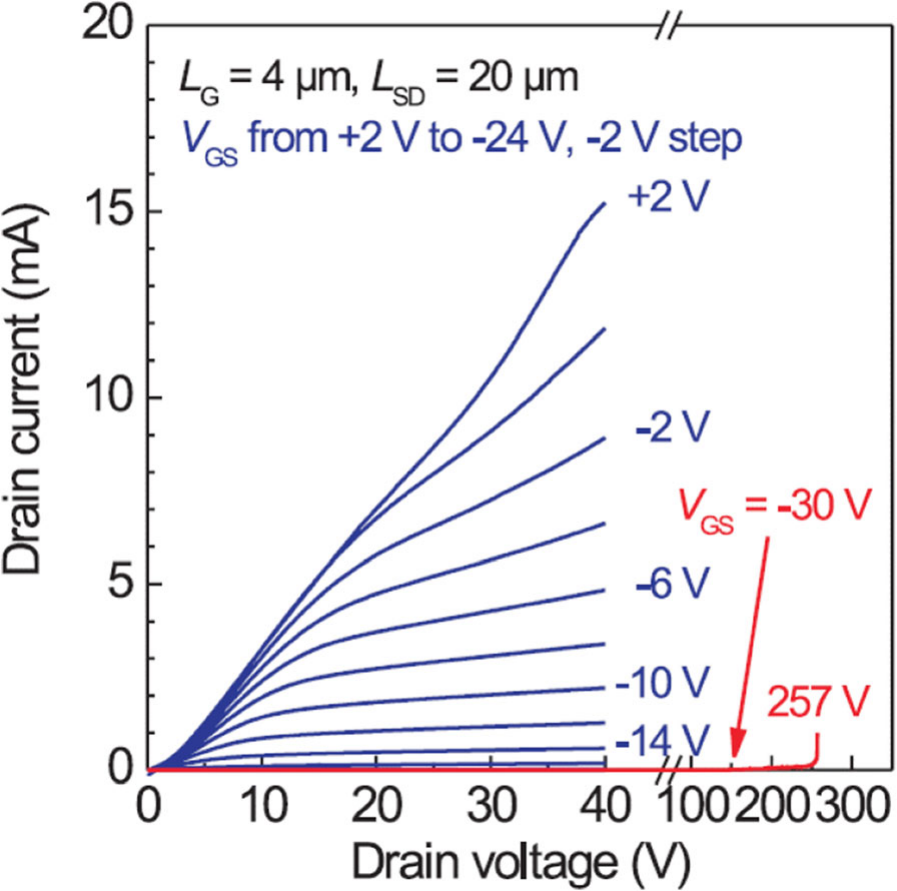
(Color online) DC output characteristics of Ga_2_O_3_ metal/semiconductor field-effect transistors. Reproduced from Ref. [1]

**Fig. 5:**
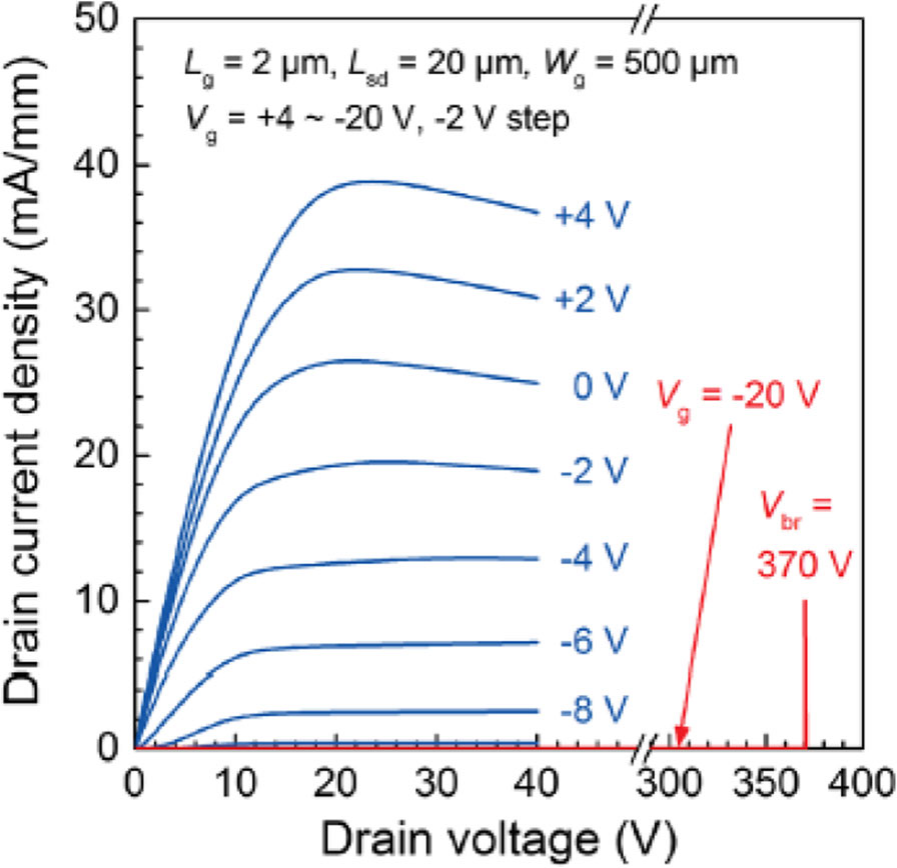
DC I–V curves of Ga_2_O_3_ MOSFET (*L*_g_ = 2 μm) measured at RT. Reproduced from Ref. [12]

The pre-treatment is performed before metal deposition, including ion implantation, plasma bombardment, and reactive-ion etching (RIE). Higashiwaki et al. demonstrated that the contacts formed by using Ti/Au stack with the RIE pre-treating process showed an almost Ohmic behavior, while the sample without the RIE treatment showed a Schottky behavior, as illustrated in Fig. 3 [1]. The significant difference could be attributed to the out-diffusing of the free oxygen atoms that generated through the continuous bombardment by breaking the exposed Ga–O bonds, leaving plenty of oxygen vacancies that act as donors in β-Ga_2_O_3_. On the other hand, the continuous RIE treatment would also generate considerable surface states which play an important role during contact formation [41]. Figure 4 shows associated DC output characteristics from which quasi-linear current at low drain voltage can be observed. In their later work, as demonstrated in Fig. 5, the output characteristics exhibited good linearity relationship between the current and drain voltage in which Si ion implantation and RIE were applied to β-Ga_2_O_3_ together and an extremely low specific contact resistivity of 8.1 × 10^−6^Ω∙cm^2^ was achieved [12]. Obviously, the Ohmic behavior obtained by RIE and Si^+^ implantation together would outperform that by RIE only since Si atoms are known to be shallow donors with small activation energies in β-Ga_2_O_3_ [34]. Additionally, Zhou et al. reported the high-performance β-Ga_2_O_3_ field-effect transistors with Ar plasma bombardment prior to contact metal deposition [52]. On the contrary, the sample without Ar bombardment exhibited Schottky contacting. The difference can be ascribed to the generation of oxygen vacancies and surface states during the Ar plasma bombardment process, the same as RIE treatment.

Although the abovementioned techniques can improve the performance of Ohmic contacts, such technologies are not practically applicable because the induced damage is usually the last thing that process engineers want in semiconductor devices, and furthermore, the damage-induced Ohmic contacts are not always reproducible.

**Fig. 6:**
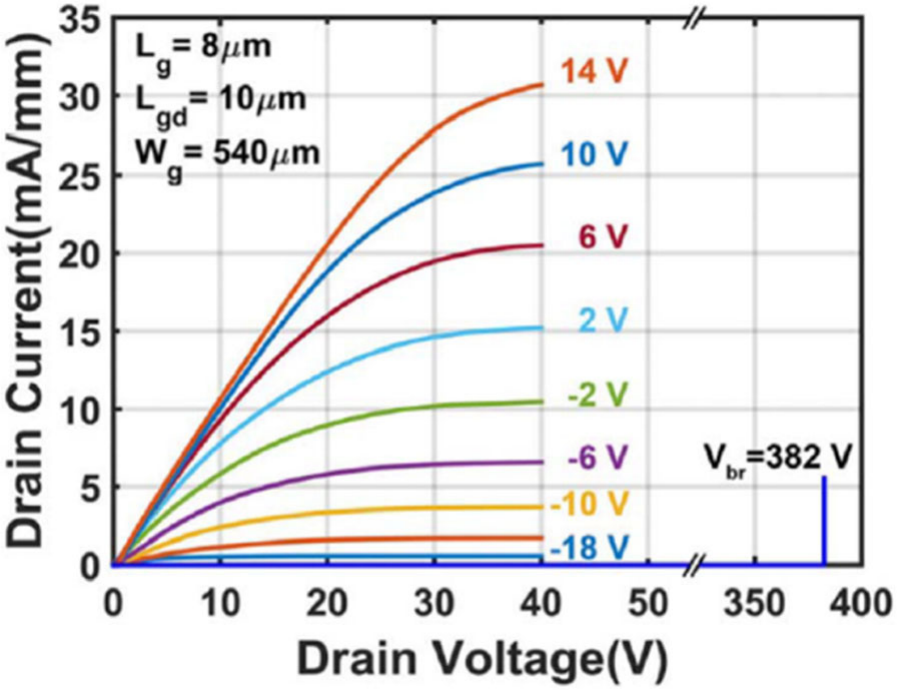
Output characteristics of the SOG S/D-doped MOSFET with *L*_g_ = 8 μm, drain gate spacing *L*_gd_ = 10 μm. Reproduced from Ref. [53]

For this reason, apart from the aforesaid traditional techniques used frequently for forming low-resistance Ohmic contacts, a relatively novel technique—spin-on-glass (SOG) doping—was recently adopted [53], and a specific contact resistivity of 2.1±1.4 × 10^−5^Ω∙cm^2^ was achieved, which verified the effectiveness of SOG doping technique. Figure 6 shows the output characteristics of SOG-doped β-Ga_2_O_3_ MOSFETs which exhibited excellent linear behavior at low drain voltage. Compared with ion implantation, the SOG doping reduces damage-induced diffusion of species and lowers the costs by abandoning the expensive ion implanter. Similar to ion implantations, the basic principle behind this technology is doping the S/D region with shallow donors. Obviously, the superior Ohmic contact can be achieved with intentionally doped β-Ga_2_O_3_. For instance, the highly doped β-Ga_2_O_3_ was used to fabricate β-Ga_2_O_3_ field-effect transistors with drain currents exceeding 1.5 A/mm [50]. The record high drain current is due to the heavy doping in β-Ga_2_O_3_ which causes a very thin depletion layer, and electrons can tunnel easily across this barrier leading to an Ohmic contacting behavior. Interestingly, the orientation of the β-Ga_2_O_3_ surface may also exert an influence on the contacting behavior. Baik et al. reported that the same electrodes on β-Ga_2_O_3_ showed different contact properties, in which the sample on ($\overline{2}$01) substrate behaved as Ohmic contacts while the control sample on (010) exhibited Schottky behavior. This could be attributed to different Ga/O ratio and density of dangling bonds at specific orientations [54].

### Post-treatment

**Fig. 7:**
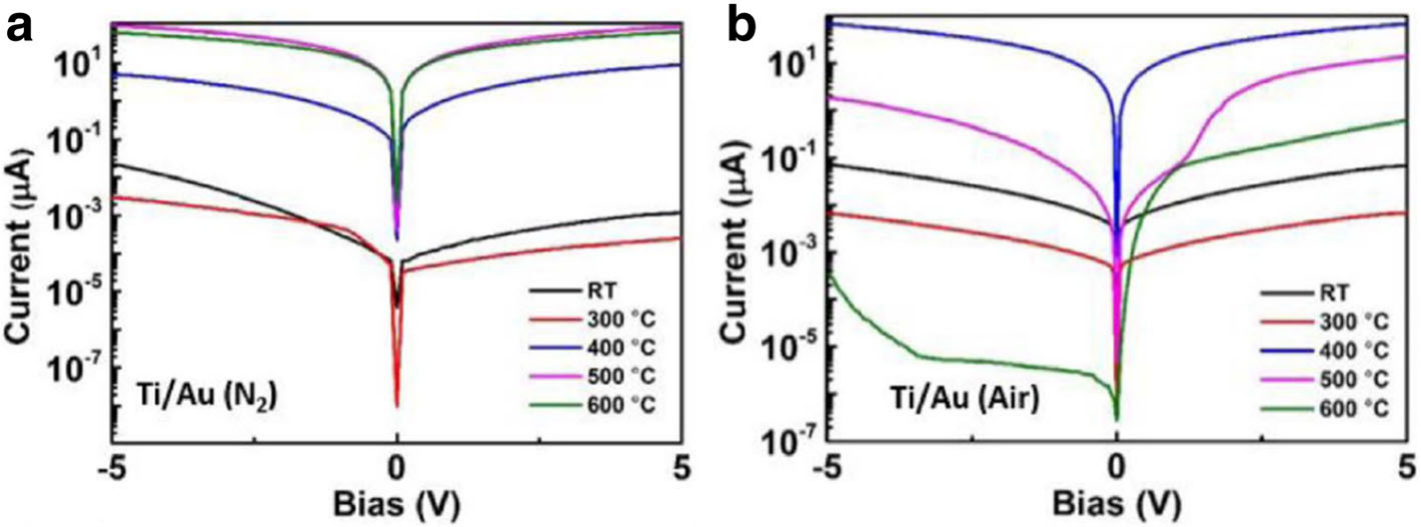
Electrical properties of β-Ga_2_O_3_ flakes with different thermal annealing atmosphere and annealing temperature. Ti/Au contacts under **a** N_2_ and **b** air. Reproduced from Ref. [55]

The post-treatment is performed after metal deposition, mainly referring to the annealing process. Annealing plays a role in reducing damage induced by previous process technologies such as ion implantation and plasma bombardment. Additionally, it contributes to the formation of an interlayer which may reduce the conduction band discontinuity between the metal and β-Ga_2_O_3_. Remarkably, the parameters including temperature, atmosphere, and annealing time exert an important influence on the performance of devices. The experiment on the annealing in air and N_2_ was implemented to compare the effect of annealing atmosphere on β-Ga_2_O_3_-based Ohmic contacts [55]. As it can be seen in Fig. 7, the performance of annealing in N_2_ outperformed that in air, which could be attributed to that higher oxygen partial pressure in air suppressed the formation of oxygen vacancies. However, the dependence of contact characteristics on the temperature, atmosphere, and annealing time on contact characteristics is unclear; hence, it is further needed to optimize the parameters of the annealing process.

### Multilayer Metal Electrode

Another approach to forming Ohmic contacts is to reduce the SBH at the metal/semiconductor interface. The SBH equals the difference between the work function of the metal and the electron affinity of the semiconductor. Based on this recognition, one might expect that metals with low work function would form Ohmic contacts on β-Ga_2_O_3_. Nevertheless, it has been proven that the work function is not the dominant factor of forming Ohmic contact [56].

**Fig. 8:**
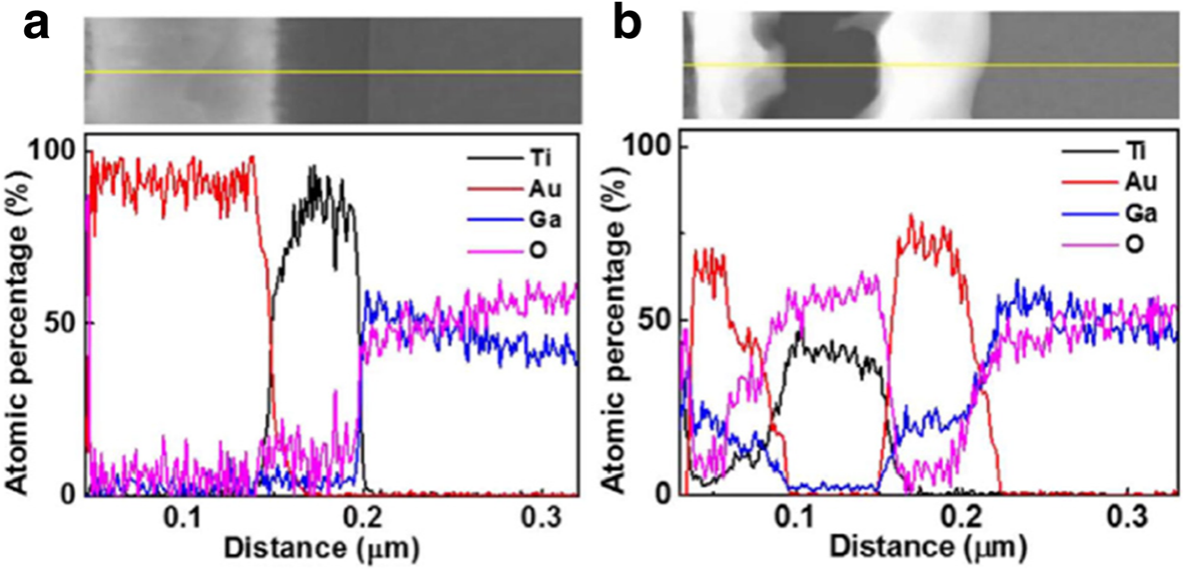
Atomic percentage profiles by EDS of metallization and β-Ga_2_O_3_ **a** pre- and **b** post-annealing at a temperature of 500 ^°^C. Reproduced from Ref. [55]

Nine metals deposited on β-Ga_2_O_3_ were selected based on the properties such as work function, melting temperature, and oxide stability [57]. The metal work function of Ti and electron affinity of unintentionally doped β-Ga_2_O_3_ are known to be 4.33 eV and 4.00±0.05 eV, respectively [19, 58, 59], so a barrier of 0.22 eV should exist at the interface leading to the Schottky contact. Nonetheless, it turned out that Ti contacts with an Au capping layer were Ohmic with the lowest resistance among nine metals after annealing. In the meanwhile, Bae et al. explored the dependence of contact properties on the Ti/Au and Ni/Au for devices based on the exfoliated β-Ga_2_O_3_ flakes [55]. It was observed that the performance of MOSFETs with Ti/Au metal electrodes outperformed those with Ni/Au metal electrodes under the same annealing condition. At the beginning, it was considered that the work functions of Ni and Ti are 5.01 eV and 4.33 eV, respectively, so Ti may form an Ohmic contact more easily than Ni; however, studies through the energy dispersive spectroscopy (EDS) demonstrated that the oxygen atomic percentage in the β-Ga_2_O_3_ region decreased while the oxygen atomic percentage in Ti near the interface increased after annealing, as illustrated in Fig. 8 [55]. This phenomenon is ascribed to the out-diffusion of oxygen atoms from β-Ga_2_O_3_ into Ti metal, leading to the formation of oxygen vacancies acting as donors. Moreover, during the annealing process, the accelerated out-diffusion of oxygen atoms in β-Ga_2_O_3_ could react with Ti and form Ti_2_O_3_ which is useful for forming Ohmic contacts owing to its low work function (3.6–3.9 eV). Therefore, the interfacial reaction between metals and β-Ga_2_O_3_ is an important factor in forming Ohmic contacts at the metal/semiconductor interface.

**Fig. 9:**
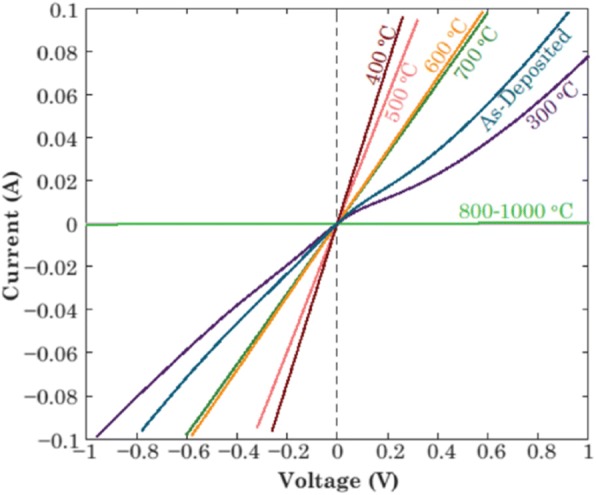
I–V plots for Ti/Au contacts on Sn-doped ($\overline{2}$01) Ga_2_O_3_ wafer as a function of annealing temperature in Ar (annealing time 1 min). Reproduced from Ref. [56]

In addition, it is found that most Ti/Au metal electrodes used to form Ohmic contacts were annealed at 450 ^°^C [45, 53] or 470 ^°^C [12, 46, 57, 60] by rapid thermal process. A similar degradation behavior of contact characteristics was observed when the annealing was performed above 500 ^°^C in Ref. [55, 56], as illustrated in Figs. 7 and 9, respectively. Yao et al. speculated that an insulating oxide layer was formed possibly at elevated annealing temperature, resulting in the deteriorated contacts. Nevertheless, Bae et al. observed that the surface of deposited metal was much rougher after 700 ^°^C annealing due to the intermixing of metals and the diffusion of gallium and oxygen atoms into metal electrodes, which was ascribed as the reason for degradation behavior. Obviously, the degradation mechanisms of Ti/Au contacts to β-Ga_2_O_3_ after high-temperature annealing are still under debate.

β-Ga_2_O_3_-based devices with Ti/Au contacts cannot meet the demand for working under high temperature. Hence, to avoid the degradation of contact characteristics at elevated annealing temperature, more complex metal stacks should be adopted. By far, Ti/Al/Au [50, 52], Ti/Au/Ni [61, 62], and Ti/Al/Ni/Au metal stacks [13, 21, 63, 64] have been employed to form electrical contacts on β-Ga_2_O_3_. But a comprehensive comparison of contact characteristics between these metal stacks is still insufficient.

**Fig. 10:**
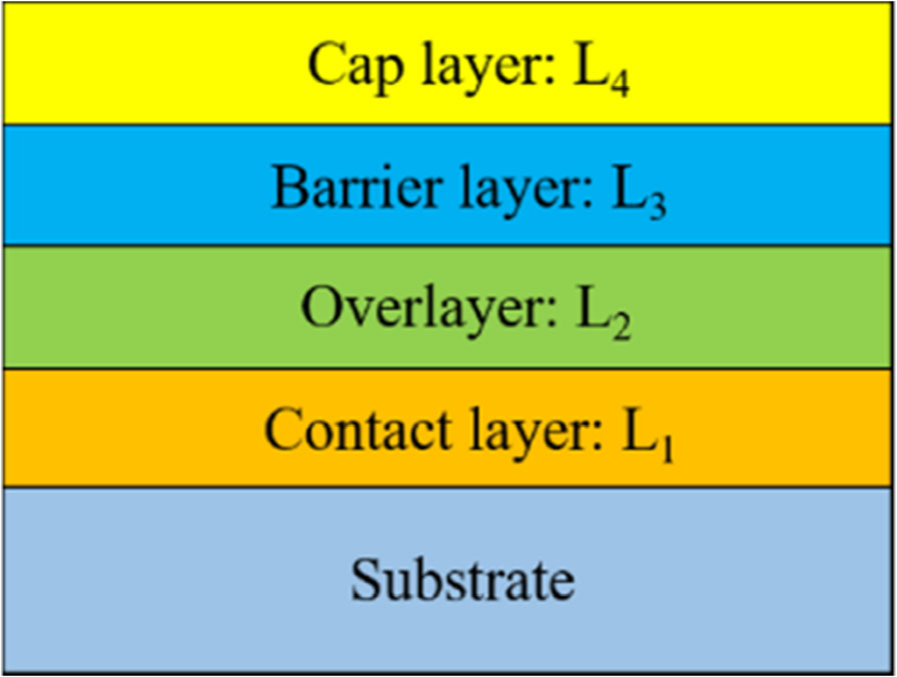
The schematic of metal stacks for obtaining Ohmic contacts to wide-bandgap semiconductors

Mohammad [65] and Greco et al. [36] discussed the role of each metal layer in the complex metal stacks, providing some guidelines for improving the Ohmic contacts. The schematic of the metal stacks is shown in Fig. 10. Note that this approach is currently developing for GaN-based power devices [66–69].

The first metal layer on the substrate, referred to as contact layer, should have a low work function and good adhesion to the substrate. Moreover, it may also block the diffusion of upper layer metals with large work functions into the substrate. Presently, Ti is the principal metal as contact layer to β-Ga_2_O_3_ because of its low function (4.33 eV) and good adhesion to the substrate. Besides, the formation of Ti_2_O_3_ and Ti_3_O_5_ with lower work functions than Ti at the interface is favored in forming Ohmic contacts since the oxides reduce the SBH and leave behind oxygen vacancies acting as donors. However, other metals with low work functions including Ta (3.1 eV) and Hf (3.9 eV) have not been explored yet. The second overlayer with a low work function should be able to form intermetallic compounds with the contact layer to prevent their diffusion into the interface. Presently, Al is used as the overlayer since it can meet these requirements. The third metal layer (barrier layer) serves the purpose of limiting the in-diffusion of the upper metal layer and out-diffusion of lower metal layers [70, 71]. Ni is the most commonly used barrier layer for β-Ga_2_O_3_. There are other good candidates like Mo, Nb, and Ir with high melting points to substitute Ni which are expected to have lower reactivity and solubility for Au than Ni [72–75]. The fourth cap layer acts as a protective layer to prevent or minimize the oxidation of underlying metals. Practically, Au is commonly employed to serve this purpose.

### Introducing an Interlayer

**Fig. 11:**
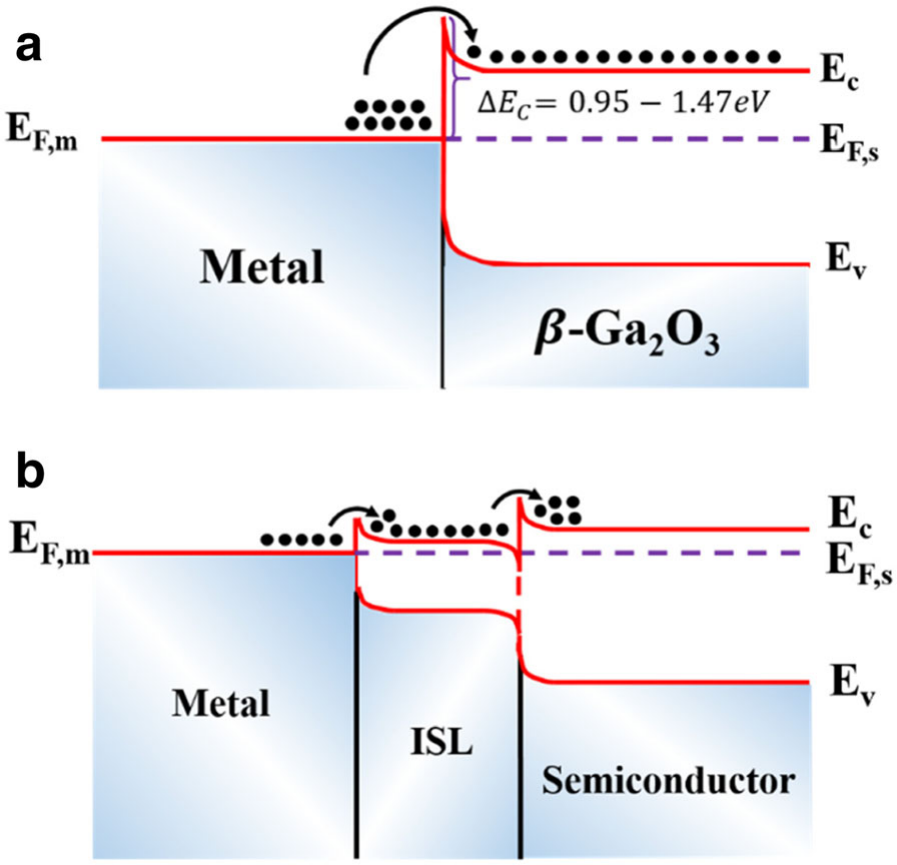
The schematic of band offsets for **a** metal/β-Ga_2_O_3_ and **b** metal/ISL/β-Ga_2_O_3_. *∆E*_*c*_ equals the energy difference between the Fermi energy of metals and the conduction band of semiconductors

There are mainly two methods of introducing an interlayer at the metal/β-Ga_2_O_3_ interface. One is to form an intermediate semiconductor layer (ISL) with low work function by annealing, e.g., Ti_2_O_3_. The other is to insert the deposited ISL between the metal and β-Ga_2_O_3_, which has been intensively studied [76–78]. Compared with the former method, the latter is more favorable to form Ohmic contacts owing to the high carrier concentration of ISL. The bandgaps of ISLs range from 3.5 to 4.0 eV [79–81], like AZO (~ 3.2 eV) [82], In_2_O_3_ (~ 2.9 eV) [83, 84], and IGZO (~ 3.5 eV) [85]. Typically, the SBHs of various metals deposited on β-Ga_2_O_3_ are in the range of 0.95–1.47 eV [86, 87], as shown in Fig. 11a. Nonetheless, the incorporation of a thin ISL reduces the SBH, making it easier for electrons to transport from the metal to the conduction band of β-Ga_2_O_3_, as illustrated in Fig. 11b. Additionally, the high density of electrons in ISL could further reduce the contact resistance.

Lately, AZO/Ti/Au was used as electrodes on Si^+^-implanted β-Ga_2_O_3_, and the obtained specific contact resistivity was 2.82 × 10^−5^ Ω∙cm^2^ after annealing [76]. Oshima et al. achieved platinum/indium–tin oxide (Pt/ITO) Ohmic contacts to β-Ga_2_O_3_ with a wide range of process temperature window [77]. The large process window of 900–1150 ^°^C enables the realization of high-temperature operation. And ITO/Ti/Au electrodes to β-Ga_2_O_3_ were also demonstrated by Carey et al. [78] in which the sample showed Ohmic behavior with *ρ*_*C*_ of 6.3 × 10^−^5 Ω∙cm^2^ after annealing. Without the ITO, the same annealing did not deliver linear current–voltage characteristics. These results verify the effectiveness of adding ISL for obtaining Ohmic contacts.

Notably, a bubble on the surface of ITO/Ti/Au contacts was observed while no bubbling on the single ITO layer without metal layer above [78]. It was considered as the result of out-diffusion of oxygen atoms in the ITO layer into the upper metal layers. Hence, it is necessary to choose appropriate metal or metal stacks as capping layers on ITO to prevent the degradation of surface morphology.

## Conclusions

In this work, we have summarized the significant progress in R&D of β-Ga_2_O_3_ MOSFETs. Nevertheless, the contacts on β-Ga_2_O_3_ are one of the key issues limiting its potential application as high-frequency and high-voltage devices in the future. Although this review provides an overview of the state-of-the-art methods for forming Ohmic contacts, there is still much space left to be explored, and a set of concise prospects can be digested as follows: (i) The temperature dependence and degradation mechanism of contact characteristics need further investigations for clear clarification; (ii) Metals with low work function like Ta and Hf and metals with high melting point like Mo, Nb, and Ir are worthy to be screened for serving as the contact layer and barrier layer, respectively; (iii) The optimal metal stacks on β-Ga_2_O_3_ have not been fully realized yet, and a comprehensive and systematic study of metal stacks to β-Ga_2_O_3_ is imperative for achieving low-resistance and thermally stable Ohmic contacts; and (iv) Other potential ISLs consisting of ZnO, IZO, IGZO, etc. remain unexploited, as well as the influence of varying thickness and proportion of ingredients of ISLs on the performance of the contacts. In summary, the studies about Ohmic contacts to β-Ga_2_O_3_ are still quite superficial; we believe that this topic will continue to be one of the focused issues in the future. Hopefully, the approaches to forming Ohmic contacts presented in this review would be instrumental in achieving high-performance β-Ga_2_O_3_ devices.
